# Sequential Organ Failure Assessment Score as a Predictor of Intensive Care Unit Mortality in Patients With Maxillofacial Trauma: A Prospective Observational Study

**DOI:** 10.7759/cureus.116925

**Published:** 2026-09-25

**Authors:** Hanish Shashidhar, Prasad P Ingley, Arani Roy, Sanpreet S Sachdev, Pritha Negi, Mohammed Taslim Fathima, Niti Prasad Ingley, Rahul VC Tiwari

**Affiliations:** 1 Department of General Medicine and Critical Care, Manorama Super Speciality Hospital, Nizamabad, IND; 2 Department of Anaesthesiology and Critical Care, Tata Main Hospital, Dhanbad, IND; 3 Department of Dentistry, Balurghat District Hospital, Balurghat, IND; 4 Department of Oral Pathology and Microbiology, Bharati Vidyapeeth (Deemed to be University) Dental College and Hospital, Mumbai, IND; 5 Department of Prosthodontics, Crown and Bridge, K. M. Shah Dental College and Hospital, Sumandeep Vidyapeeth (Deemed to be University), Vadodara, IND; 6 Department of Oral and Maxillofacial Surgery, Care Dental College, Guntur, IND; 7 Department of Otolaryngology, Tata Main Hospital, Dhanbad, IND; 8 Department of Oral and Maxillofacial Surgery, RKDF Dental College and Research Centre, Bhopal, IND

**Keywords:** intensive care units, maxillofacial injuries, mortality, multiple organ failure, prognosis

## Abstract

Introduction: Maxillofacial trauma that requires intensive care may be complicated by systemic organ dysfunction and mortality. The Sequential Organ Failure Assessment (SOFA) score provides an objective measure of physiological deterioration, whereas serial assessments may offer greater prognostic information than a single measurement. This study evaluated the prognostic performance of the admission SOFA score, 48-hour SOFA score, and ΔSOFA score (ΔSOFA) for predicting intensive care unit (ICU) mortality.

Materials and methods: This prospective observational study included 130 consecutive patients with maxillofacial trauma admitted to the ICU over a one-year period from June 2018 to May 2019. SOFA scores were recorded at admission to the ICU (SOFA-0) and at 48 hours (SOFA-48). ΔSOFA was calculated as SOFA-48 minus SOFA-0. The patients were categorized according to their ICU survival status. Receiver operating characteristic (ROC) curve analysis was performed to evaluate the discriminatory ability of each SOFA score.

Results: Of 130 patients, 105 survived, and 25 died during ICU admission. Non-survivors had higher SOFA-0 scores than survivors (11.0 ± 3.5 vs. 6.5 ± 3.0; p < 0.001) and higher SOFA-48 scores (13.8 ± 4.2 vs. 5.2 ± 3.0; p < 0.001). ΔSOFA demonstrated worsening organ dysfunction among non-survivors compared with improvement among survivors (2.8 ± 3.6 vs. −1.3 ± 2.5; p < 0.001). ROC analysis showed an area under the curve (AUCs) of 0.79 for SOFA-0, 0.91 for SOFA-48, and 0.93 for ΔSOFA. A ΔSOFA cut-off of >+1.5 demonstrated 92.0% sensitivity and 81.0% specificity.

Conclusion: Serial SOFA assessment, particularly ΔSOFA, demonstrated strong prognostic utility for ICU mortality in patients with maxillofacial trauma. Early worsening of organ dysfunction may help identify patients at increased risk of poor outcomes.

## Introduction

Maxillofacial trauma is frequently associated with severe systemic injury, airway compromise, neurological impairment, and multiple organ dysfunction, particularly in patients who require intensive care [1]. Although anatomical injury severity is important for initial management, clinical outcomes in critically ill trauma patients are strongly influenced by the development and progression of organ dysfunction [2]. Therefore, the early identification of patients at an increased risk of mortality may facilitate the timely escalation of monitoring and supportive treatment.

The Sequential Organ Failure Assessment (SOFA) score provides a standardized assessment of dysfunction across six organ systems and is widely used in critical care to quantify the severity of physiological derangement [3]. However, a single SOFA measurement at admission may not adequately reflect the subsequent clinical trajectory [4]. Serial assessment, particularly evaluation of the change in the SOFA score (ΔSOFA) during the first 48 hours, may provide additional prognostic information because improvement or deterioration of organ function can indicate the patient's response to critical illness and treatment [5].

In patients with maxillofacial trauma requiring intensive care, evidence regarding the comparative prognostic performance of the admission SOFA, 48-hour SOFA, and dynamic ΔSOFA scores remains limited [6]. Therefore, the present study evaluated these three measures in relation to intensive care unit (ICU) mortality. The primary aim of this study was to evaluate and compare the predictive performance of the SOFA score at ICU admission (SOFA-0), at 48 hours (SOFA-48), and the change in SOFA score over 48 hours (ΔSOFA) in predicting ICU mortality among patients with maxillofacial trauma. The secondary objectives were to compare SOFA-0, SOFA-48, and ΔSOFA between survivors and non-survivors; to assess the association between changes in SOFA score during the first 48 hours and ICU mortality; and to determine the discriminatory ability, optimal cut-off values, sensitivity, and specificity of SOFA-0, SOFA-48, and ΔSOFA for predicting ICU mortality using receiver operating characteristic (ROC) curve analysis.

## Materials and methods

Study design and setting

This prospective observational study was conducted in the Department of Oral and Maxillofacial Surgery, RKDF Dental College and Research Centre, Bhopal, India, over a period of one year, from June 2018 to May 2019. The study population comprised consecutive patients with maxillofacial trauma who were admitted to the ICU during the study period. The study was observational in design, and no alterations were made to the treatment provided by the treating clinicians based on study participation.

Ethical considerations

This study was initiated after obtaining approval from the ethics committee (Ref. no: RKDF/DC/2018/SS-28) of the study institution. The study was conducted in accordance with the ethical principles governing research involving human participants, and confidentiality of patient information was maintained throughout the study. Written informed consent was obtained from the patient or, when the patient's clinical condition precluded informed consent, from an appropriate legally authorized representative, as applicable. Patient identifiers were removed from the analytical dataset, and the collected information was used exclusively for research purposes.

Study population and eligibility

Consecutive patients with clinically diagnosed maxillofacial trauma who required ICU admission during the study period were included. Patients were considered eligible if they had sustained maxillofacial trauma and required intensive care management with sufficient clinical information to calculate the SOFA score at ICU admission and again at 48 hours. Patients without adequate information to calculate either of the predefined SOFA measurements were excluded from the analysis. The final analytical cohort comprised 130 patients. Patients were included only if the SOFA score could be calculated at ICU admission and again at 48 hours. Patients who died, were discharged from the ICU, or were transferred before 48 hours, and those with incomplete data for either time point, were excluded. No imputation of missing 48-hour scores was performed. SOFA-48 and ΔSOFA were therefore analysed as landmark measures among patients who remained in the ICU and were assessable at 48 hours.

Clinical data collection

Demographic and clinical information was extracted from available medical records and ICU documentation. The recorded variables included age, sex, mechanism of trauma, presence of associated injuries, Glasgow Coma Scale score at admission [7], endotracheal intubation, surgical intervention, duration of ICU stay, and duration of mechanical ventilation. Associated injuries were recorded as present or absent based on the available clinical documentation. Detailed injury-specific classifications, formal Injury Severity Scores, standardized facial injury severity scores, and specific indications for ICU admission were not consistently available across the retrospective records and were therefore not included in the analysis. Patients were subsequently categorized according to ICU outcome as survivors or non-survivors. The primary outcome was ICU mortality.

ICU length of stay was calculated from ICU admission to ICU death or ICU discharge. Duration of mechanical ventilation was defined as the total duration of invasive ventilation during the index ICU admission only; ventilation in the emergency department or operating theatre before ICU admission, and ventilation after ICU discharge, were not included. Patients who were never invasively ventilated were counted in the ventilation prevalence row but were excluded from the ventilation-duration summary.

Assessment of SOFA score

The SOFA score was calculated according to the original criteria described by Vincent et al. [8]. The score evaluates six organ systems-respiratory, coagulation, hepatic, cardiovascular, neurological, and renal-with each component assigned a score from 0 to 4, giving a total score ranging from 0 to 24. SOFA was calculated at two predefined time points: immediately following ICU admission (SOFA-0) and 48 hours after ICU admission (SOFA-48), using the clinical and laboratory parameters documented at the respective assessment time points. The change in SOFA score over the first 48 hours was calculated for each patient as ΔSOFA = SOFA-48 − SOFA-0. A negative ΔSOFA indicated improvement in overall organ function during the first 48 hours, whereas a positive ΔSOFA indicated worsening organ dysfunction. SOFA-0, SOFA-48, and ΔSOFA were prespecified predictor variables evaluated in relation to ICU mortality. The prediction time origin for SOFA-0 was ICU admission, with ICU mortality assessed over the remainder of the ICU stay. For SOFA-48 and ΔSOFA, the prediction time origin was 48 hours after ICU admission; therefore, these measures were considered conditional prognostic measures available at 48 hours, with ICU mortality assessed from 48 hours onward until ICU discharge or death.

Sample size

Because the study included all consecutive eligible patients with maxillofacial trauma admitted to the ICU during the predefined one-year study period, an a priori sample-size calculation was not performed. The final analytical cohort comprised 130 patients. The magnitude and precision of the observed between-group differences were assessed using effect sizes and corresponding 95% confidence intervals (CIs).

Statistical analysis

All statistical analyses were performed using Statistical Product and Service Solutions (SPSS, version 26.0; IBM SPSS Statistics for Windows, Armonk, NY). The distribution of continuous variables was assessed using the Shapiro-Wilk test. Normally distributed continuous variables were expressed as mean ± standard deviation (SD), whereas non-normally distributed variables were summarized as medians with interquartile ranges (IQR). Categorical variables were presented as frequencies and percentages. For comparisons between ICU survivors and non-survivors, normally distributed continuous variables were compared using the independent-samples t-test, whereas the Mann-Whitney U test was used for non-normally distributed continuous variables. Categorical variables were compared using Pearson's chi-square test, and Fisher's exact test was used when the assumptions of the chi-square test were not satisfied.

Effect sizes were reported alongside the hypothesis testing results to quantify the magnitude of between-group differences. Cohen's d was used for independent group continuous comparisons based on parametric analysis, rank-biserial correlation (r) for non-parametric comparisons, and Cramér's V for categorical comparisons. Corresponding 95% CIs were reported where applicable. The predictive performance of SOFA-0, SOFA-48, and ΔSOFA for ICU mortality was evaluated using a ROC curve analysis. The area under the ROC curve (AUC) with 95% CI was calculated for each predictor. The statistical significance of each AUC was assessed, and the corresponding Z-statistic was reported. The optimal cut-off value was determined using the Youden index. The sensitivity and specificity with 95% CIs were reported as the optimal cut-off values for each SOFA measure. The discriminatory performance of the three predictors was then compared descriptively to determine whether dynamic changes in SOFA provided greater prognostic discrimination than either admission or 48-hour SOFA alone. Statistical significance was defined as a two-sided p < 0.05.

The primary analysis focused on the prognostic and discriminatory performance of SOFA-0, SOFA-48, and ΔSOFA in distinguishing ICU survivors from non-survivors. Because the study was designed to evaluate the prognostic performance of these prespecified SOFA measures rather than to estimate an independent causal association with mortality, the primary analyses comprised unadjusted between-group comparisons and ROC curve analyses.

## Results

During the study period, 158 patients with maxillofacial trauma were admitted to the ICU and screened. Of these, 28 were excluded: 9 died before 48 hours, 14 were discharged or transferred from the ICU before 48 hours, and 5 had insufficient data to calculate SOFA-0 and/or SOFA-48. The remaining 130 patients were alive and assessable in the ICU at 48 hours and constituted the analytical cohort. Of these, 105 (80.8%) survived to ICU discharge and 25 (19.2%) died in the ICU; all 25 deaths occurred after the 48-hour landmark. Of these, 105 (80.8%) were survivors, and 25 (19.2%) were non-survivors. The baseline demographic and clinical characteristics of the study population according to ICU outcomes are presented in Table 1.

**Table 1:** Baseline demographic and clinical characteristics of survivors and non-survivors. Data are presented as mean ± standard deviation (SD) for normally distributed continuous variables, median (interquartile range (IQR)) for non-normally distributed continuous variables, and frequency (percentage) for categorical variables. Between-group comparisons were performed using the independent-samples t-test for normally distributed continuous variables, the Mann-Whitney U test for non-normally distributed continuous variables, and the Pearson chi-square test for categorical variables, as appropriate. Effect sizes are reported as Cohen's d for parametric continuous comparisons, rank-biserial correlation (r) for non-parametric comparisons, and Cramér's V for categorical comparisons. ICU length of stay was calculated from ICU admission to ICU death or ICU discharge. Duration of mechanical ventilation was restricted to invasive ventilation during the index ICU admission and was summarised only among ventilated patients. *p < 0.05 was considered statistically significant. GCS: Glasgow Coma Scale; ICU: intensive care unit; df: degrees of freedom

Characteristic		Survivors, n = 105 (80.8%)	Non-survivors, n = 25 (19.2%)	Test statistic (df)	p-value	Effect size
Age (years)	Mean ± SD	41.2 ± 16.5	58.4 ± 14.8	t (128) = 4.82	< 0.001*	d = 1.06 (0.62-1.50)
Sex (male)	N (%)	80 (76.2%)	17 (68.0%)	χ² (1) = 0.68	0.409	V = 0.07 (0.00-0.22)
Mechanism of trauma						
Road traffic accident	N (%)	68 (64.8%)	14 (56.0%)	χ² (2) = 1.19	0.551	V = 0.10 (0.00-0.25)
Fall from height	N (%)	23 (21.9%)	6 (24.0%)			
Assault	N (%)	14 (13.3%)	5 (20.0%)			
Associated injuries	N (%)	63 (60.0%)	19 (76.0%)	χ² (1) = 2.11	0.146	V = 0.13 (0.00-0.27)
GCS at admission	Median (IQR)	11 (9-14)	6 (5-8)	U = 425.5	< 0.001*	r = 0.49 (0.34-0.62)
Endotracheal intubation	N (%)	42 (40.0%)	20 (80.0%)	χ² (1) = 12.65	< 0.001*	V = 0.31 (0.15-0.46)
Surgical intervention	N (%)	78 (74.3%)	14 (56.0%)	χ² (1) = 3.13	0.077	V = 0.16 (0.00-0.30)
ICU length of stay (days)	Median (IQR)	9 (6-15)	3 (2-5)	U = 390.0	< 0.001*	r = 0.52 (0.38-0.64)
Duration of mechanical ventilation (days), ventilated patients only	Median (IQR)	4 (1-8)	12 (5-16)	U = 580.5	< 0.001*	r = 0.38 (0.22-0.52)
Mechanical ventilation (n, %)	N (%)	52 (49.5%)	20 (80.0%)	χ² (1) = 7.47	0.006*	V = 0.24 (0.08-0.39)

Non-survivors were significantly older than survivors (58.4 ± 14.8 vs. 41.2 ± 16.5 years; p < 0.001) and had significantly lower admission GCS scores (median: 6 (IQR: 5-8) vs. 11 (IQR: 9-14); p < 0.001). Endotracheal intubation was significantly more frequent among non-survivors (80.0% vs. 40.0%; p < 0.001). Non-survivors had a significantly shorter ICU stay (median: 3 (IQR: 2-5) vs. 9 (IQR: 6-15) days; p < 0.001). Mechanical ventilation was required in 80.0% of non-survivors compared with 49.5% of survivors (p = 0.006). Among ventilated patients, median duration of invasive ventilation during the ICU stay was 3 (IQR: 2-4) days in non-survivors and 4 (IQR: 2-7) days in survivors. No significant differences were observed for sex, mechanism of trauma, associated injuries, or surgical intervention (Table 1).

Comparison of SOFA scores

A significant difference in SOFA scores was observed between survivors and non-survivors (Table 2). The mean SOFA-0 score was significantly higher in non-survivors than in survivors (11.0 ± 3.5 vs. 6.5 ± 3.0; p < 0.001; Cohen's d = 1.45, 95% CI: 1.00-1.90). The difference was even greater for SOFA-48, with non-survivors demonstrating a substantially higher score than survivors (13.8 ± 4.2 vs. 5.2 ± 3.0; p < 0.001; Cohen's d = 2.39, 95% CI: 1.85-2.93). The change in SOFA score over the first 48 h also differed significantly between the two groups. Survivors demonstrated an improvement in organ function, with a mean ΔSOFA of −1.3 ± 2.5, whereas non-survivors demonstrated worsening organ dysfunction, with a mean ΔSOFA of +2.8 ± 3.6 (p < 0.001). The corresponding rank-biserial effect size was r = 0.57 (95% CI: 0.44-0.68) (Table 2).

**Table 2:** Comparison of SOFA scores between survivors and non-survivors. Data are presented as mean ± SD for SOFA-0, SOFA-48, and ΔSOFA. Between-group comparisons were performed using the independent-samples t-test for SOFA-0 and SOFA-48 and the Mann-Whitney U test for ΔSOFA. Effect sizes are presented as Cohen's d for parametric comparisons and rank-biserial correlation (r) for non-parametric comparisons, with 95% confidence intervals (CI). *p < 0.05 was considered statistically significant. SOFA-0: Sequential Organ Failure Assessment score at ICU admission; SOFA-48: Sequential Organ Failure Assessment score at 48 hours; ΔSOFA: change in SOFA score between 48 hours and admission; ICU: intensive care unit

Variable	Survivors, n = 105 (80.8%)	Non-survivors, n = 25 (19.2%)	Test statistic (df)	p-value	Effect size (95% CI)
SOFA-0, Mean ± SD	6.5 ± 3.0	11.0 ± 3.5	t (128) = 6.31	< 0.001*	d = 1.45 (1.00-1.90)
SOFA-48, Mean ± SD	5.2 ± 3.0	13.8 ± 4.2	t (128) = 10.59	< 0.001*	d = 2.39 (1.85-2.93)
ΔSOFA (48 - 0), Mean ± SD	-1.3 ± 2.5	+2.8 ± 3.6	U = 315.0	< 0.001*	r = 0.57 (0.44-0.68)

ROC curve analysis

The predictive performance of the SOFA-0, SOFA-48, and ΔSOFA scores for ICU mortality was assessed using ROC curve analysis. All three measures demonstrated statistically significant discriminatory ability. SOFA-0 yielded an AUC of 0.79 (95% CI: 0.69-0.87; p < 0.001). The optimal cut-off was >9.5, providing a sensitivity of 76.0% (95% CI: 54.9-90.6) and specificity of 74.3% (95% CI: 64.9-82.3). The discriminatory performance was substantially higher for SOFA-48, which demonstrated an AUC of 0.91 (95% CI: 0.85-0.97; p < 0.001). At the optimal cut-off of >10.5, the sensitivity and specificity were 88.0% (95% CI: 68.8-97.5) and 84.8% (95% CI: 76.4-91.0). ΔSOFA demonstrated the highest observed discriminatory performance, with an AUC of 0.93 (95% CI: 0.88-0.98). The optimal cut-off was >+1.5, corresponding to a sensitivity of 92.0% (95% CI: 74.0-99.0) and specificity of 81.0% (95% CI: 72.2-87.9). The Youden index was 0.725. Thus, an increase in SOFA score of more than 1.5 points during the first 48 hours provided the strongest discrimination for ICU mortality (Table 3, Figure 1).

**Table 3:** Receiver operating characteristic (ROC) curve analysis of SOFA measures for predicting ICU mortality. ROC curve analysis was performed to assess the discriminatory ability of SOFA-0, SOFA-48, and ΔSOFA for ICU mortality. Optimal cut-off values were determined using the Youden index. *p < 0.05 was considered statistically significant. AUC: area under the curve; CI: confidence interval; SOFA-0: Sequential Organ Failure Assessment score at ICU admission; SOFA-48: Sequential Organ Failure Assessment score at 48 hours; ΔSOFA: change in SOFA score between 48 hours and admission; ICU: intensive care unit

Variable	AUC (95% CI)	p-value	Z-statistic	Optimal cut-off	Sensitivity (95% CI)	Specificity (95% CI)	Youden's index
SOFA-0	0.79 (0.69-0.87)	< 0.001*	7.81	> 9.5	76.0% (54.9-90.6)	74.3% (64.9-82.3)	0.486
SOFA-48	0.91 (0.85-0.97)	< 0.001*	16.42	> 10.5	88.0% (68.8-97.5)	84.8% (76.4-91.0)	0.698
ΔSOFA	0.93 (0.88-0.98)	< 0.001*	19.56	> +1.5	92.0% (74.0-99.0)	81.0% (72.2-87.9)	0.725

**Figure 1:**
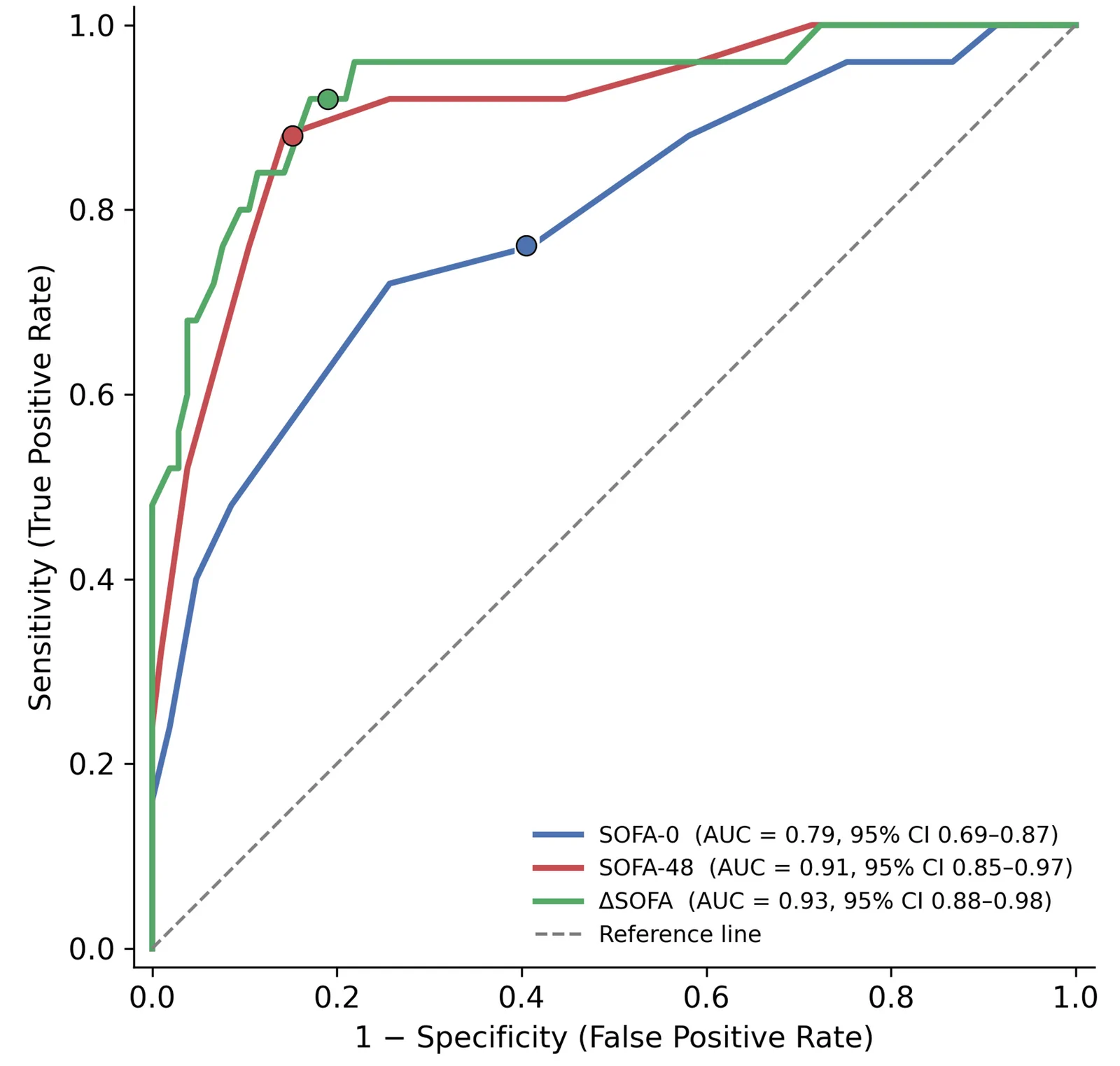
Receiver operating characteristic curves for SOFA-0, SOFA-48, and ΔSOFA in predicting ICU mortality. SOFA-0: Sequential Organ Failure Assessment score at ICU admission; SOFA-48: Sequential Organ Failure Assessment score at 48 hours; ΔSOFA: change in SOFA score between 48 hours and admission; ICU: intensive care unit

## Discussion

The present study evaluated the prognostic utility of the SOFA-0 score, SOFA-48 score, and change in SOFA score over the first 48 hours among critically ill patients with maxillofacial trauma. The principal finding was that both the absolute SOFA score and its early trajectory were associated with ICU outcomes. Patients who died demonstrated greater organ dysfunction at admission, greater deterioration during the initial ICU period, and a less favorable clinical course than survivors. These findings support the idea that physiological deterioration and evolving organ dysfunction may provide prognostic information beyond the anatomical characteristics of maxillofacial trauma.

The higher SOFA-0 score among non-survivors indicated that the mortality risk was reflected in the degree of systemic physiological disturbance at ICU entry. Severe maxillofacial trauma can compromise the airway and may coexist with traumatic brain injury, blood loss, aspiration, hypoxemia, circulatory instability, and other systemic injuries [9]. These complications can affect several SOFA domains simultaneously, particularly respiratory, cardiovascular, and neurological function [8,10]. Similarly, a previous study demonstrated that non-survivors had higher SOFA-0 scores and greater dysfunction in the respiratory, cardiovascular, and neurological systems, supporting the applicability of SOFA in trauma populations [11].

The greater prognostic separation observed with the SOFA-48 suggests that the patient's evolving physiological response may be more informative than the initial assessment alone. The early post-traumatic period is critical for the evolution of organ dysfunction, as the physiological consequences of hemorrhage and shock, respiratory and cardiovascular dysfunction, and associated neurological injury may become evident or worsen during the initial phase of critical care [12,13]. Therefore, serial SOFA assessment during this period can capture the direction of organ dysfunction rather than relying solely on the initial severity of the illness. This interpretation is consistent with that of Ferreira et al. [5], who demonstrated that sequential SOFA assessment during the early ICU period was strongly associated with outcomes and that changes during the first 48 hours were particularly informative.

The most important finding was that ΔSOFA demonstrated the highest observed discriminatory performance among the three SOFA measures. Survivors tended to demonstrate improvements in organ function, whereas non-survivors showed progressive deterioration. This pattern has an important biological rationale: a patient's initial severity may be partly reversible after airway stabilization, hemodynamic resuscitation, ventilation, surgery, and other supportive measures, whereas continued deterioration despite intensive care may reflect persistent or progressive systemic injury [14]. Therefore, the direction of change in SOFA can provide an early indication of treatment response and physiological reserve [15]. Earlier evidence has also shown that an increasing SOFA trajectory is associated with poorer outcomes, whereas decreasing scores are associated with substantially better survival [15,16].

Our findings are also consistent with broader evidence supporting dynamic SOFA assessment. A systematic review of SOFA-based mortality prediction models found that sequential SOFA derivatives provide clinically useful prognostic information, although performance varies according to the population and derivative used [17]. Furthermore, a meta-regression of clinical trials found that ΔSOFA was more consistently associated with mortality than fixed-day SOFA measurement, supporting the concept that trajectory may be more informative than an isolated score [18]. More recent trauma data likewise suggest that SOFA can outperform purely anatomical injury scores for mortality prediction in critically ill trauma populations, emphasizing the importance of systemic physiological dysfunction in determining outcome [19].

The association between poor outcomes and a greater requirement for intubation and mechanical ventilation observed in the present study further supports the clinical relevance of these findings. Patients with severe maxillofacial trauma frequently have airway obstruction, facial swelling, impaired consciousness, aspiration risk, or associated thoracic and neurological injuries [1]. Such factors can contribute to respiratory and neurological organ dysfunction and consequently increase SOFA scores. Therefore, the observed relationship should be interpreted as reflecting the overall physiological severity of critically ill trauma, rather than the severity of maxillofacial injury in isolation.

Clinical implications and limitations

Clinically, these findings suggest that the SOFA score should not be viewed solely as an admission severity score in patients with severe maxillofacial trauma. Reassessment during the early ICU period may provide a simple and objective method for identifying patients whose organ function is improving compared with deteriorating organ function. In particular, a worsening ΔSOFA score may prompt closer hemodynamic and respiratory monitoring, reassessment of airway and neurological status, optimization of organ support, and earlier multidisciplinary intervention. However, SOFA should complement rather than replace clinical assessment and trauma-specific evaluation [20].

This study has several limitations. First, it was conducted at a single center with a relatively modest sample size and within a defined study period, which may limit the generalizability of the findings to other institutions and trauma populations. Second, the observational design precludes causal inference, and residual confounding by the severity and type of maxillofacial and associated injuries, treatment strategies, and comorbidities cannot be excluded. Detailed characterization of associated injuries, formal injury severity scores, standardized measures of facial injury severity, and specific indications for ICU admission were not consistently available in the retrospective records; therefore, the independent contribution of maxillofacial injury severity to mortality could not be assessed. Third, the analysis was limited to SOFA measurements at admission and 48 hours and did not evaluate later SOFA trajectories or other established trauma severity scores.

The substantial differences in age, admission neurological status, intubation, and mechanical ventilation between survivors and non-survivors indicate that SOFA measures may partly reflect the overall severity of critical illness rather than an isolated effect of maxillofacial trauma. Furthermore, the retrospective dataset did not consistently provide detailed characterization of associated traumatic injuries or formal trauma severity scores. Therefore, residual confounding cannot be excluded, and the observed prognostic associations should not be interpreted as independent effects of SOFA after adjustment for baseline trauma severity. The optimal SOFA cut-off values were derived and evaluated within the same cohort and were based on a relatively small number of ICU deaths (n = 25). Accordingly, these thresholds should be considered exploratory and may overestimate predictive performance in other populations. Independent validation in larger, multicenter cohorts is required before these cut-off values can be considered for clinical application. Prognostic estimates for SOFA-48 and ΔSOFA apply only to patients who remained in the ICU and were assessable at 48 hours; early deaths and early discharges were excluded by design. Finally, the prognostic thresholds identified in this single-center cohort require external validation in larger, multicenter studies before they can be routinely applied to clinical decision-making.

## Conclusions

Serial SOFA assessments provided useful prognostic information in critically ill patients with maxillofacial trauma. SOFA-0 reflected initial physiological severity, while SOFA-48 and ΔSOFA provided additional prognostic information regarding the clinical course over the first 48 hours. Worsening organ dysfunction during the first 48 hours was associated with higher ICU mortality, whereas improving SOFA trajectories were associated with more favorable ICU outcomes. Because SOFA-48 and ΔSOFA are available only after 48 hours, they should be interpreted as conditional prognostic measures rather than admission-time predictors. The observed cut-off values are exploratory and require independent validation in larger, multicenter cohorts before clinical application.
